# Meeting the needs of women who use drugs and alcohol in North-east India – a challenge for HIV prevention services

**DOI:** 10.1186/1471-2458-12-825

**Published:** 2012-09-26

**Authors:** Michelle Kermode, Chinzaning Hangzo Songput, Collins Z Sono, Temjen Nungsang Jamir, Alex Devine

**Affiliations:** 1 Nossal Institute for Global Health, University of Melbourne, 161 Barry St Carlton, Melbourne, Victoria, 3010, Australia; 2 Project ORCHID, CBCNEI Mission Compound, Panbazar, Guwahati, Assam, 781001, India

**Keywords:** Alcohol, Injecting drug use, Women, India, Health service needs

## Abstract

**Background:**

The North-east Indian states of Manipur and Nagaland consistently report relatively high HIV prevalence. The targeted HIV prevention interventions in these two states are mostly delivered by non-government organizations (NGOs), and prevention of HIV transmission by injecting drug use is their main focus. Most injecting drug users (IDUs) are male, and the services are primarily tailored to meet their needs, which are not necessarily the same as those for women. This qualitative study describes the health service needs of women who use drugs and alcohol in Manipur and Nagaland, with the goal of identifying strategies and activities that can be implemented by NGOs wanting to improve their reach among vulnerable women.

**Methods:**

In 2009-10, semi-structured in-depth interviews were conducted with 27 key informants and nine focus group discussions (FGDs) with women who use drugs and alcohol, and two FGDs with male IDUs. The thematic areas covered included: the context of female drug and alcohol use; drug and alcohol use patterns; HIV risk behaviours; barriers and facilitators of service use; perceived health needs; and expressed health service needs. The data were recorded, transcribed, translated and thematically analysed.

**Results:**

The most problematic substance for women from Nagaland was alcohol, and for women from Manipur it was heroin. The most commonly identified health problems were primarily related to the women’s drug and alcohol use, reproductive health and mental health. Other problems of major concern included social exclusion, violence, children’s welfare, and financial difficulties. The expressed service needs of these women were women-only integrated health services, women-only detoxification and rehabilitation services, mental health services, desensitization of mainstream health workers, free access to medicines, assistance to meet basic needs, and a safe place for engaging in sex work.

**Conclusion:**

The expressed health and other service needs of women who use drugs and alcohol in Manipur and Nagaland do not match the services currently provided by HIV prevention NGOs, and this may, in part, account for the relatively poor uptake of these services by women. Strategies and activities that can be implemented by NGOs to strengthen their reach to vulnerable women are identified. However, many of these women’s needs are beyond the scope of services typically offered by HIV prevention NGOs, and require a coordinated multi-sectoral response.

## Background

Research and program responses to the dual public health problems of HIV and drug use primarily focus on men and injecting drug use, with the result that there is a substantial body of evidence regarding HIV risks for male injectors. Much less is known about the nexus between non-injecting drug and alcohol use and HIV risk behaviours. This bias is partially understandable given the epidemiology of injecting drug use, which is predominantly engaged in by men, and a relatively efficient route of HIV transmission. However, between 10-40% of injecting drug users (IDUs) are women, most of whom are more vulnerable to HIV infection compared to males for a number of reasons, in particular their participation in sex work [1-3]. HIV prevention programs targeting IDUs tend to be male dominated and not very accessible for female IDUs, whose needs are not the same as their male counterparts [1,2]. Similarly, these services do not cover women who use non-injecting drugs and alcohol, unless they identify as female sex workers (FSWs), even though there is a growing awareness of the association between alcohol use and HIV risks [4-6].

Amplification of the risk of HIV infection due to the overlap between female injecting drug use and sex work is well documented both in India and elsewhere [7]. Female IDUs have riskier and more sex due to the need to earn sufficient income to procure drugs and support their basic needs, and they are sometimes expected to earn enough to support the drug use of their male partners as well [2,3,7-12]. Female IDUs’ engagement in sex work is associated with higher rates of sexually transmitted infections (STIs) that in turn increase their risk of HIV infection [10]. Women who are dependent on non-injecting drugs and alcohol are also likely to have increased vulnerability to STIs including HIV [8,13,14], but unless they are injecting or engaging in sex work, are unlikely to come to the attention of HIV prevention programs.

Women substance users are more stigmatised than their male counterparts, and this directly affects their ability to access services. They delay seeking health care because they fear judgement, and when they do attend services, the quality of the care they receive is often sub-optimal due to the negative attitudes of the health care workers and their lack of knowledge and understanding about the health and social issues linked with female substance use [1,2,7,14-17]. Women dependent on substances are less likely, over their lifetime, to enter treatment compared to men. However, gender does not predict treatment retention, completion, or outcome [1,18]. Women are more vulnerable to the adverse social and mental health consequences of substance use including depression, insomnia, anxiety, post-traumatic stress disorder, suicidal tendencies, poor self-image, reduced family support, family rejection, and guilt for neglecting children [1,7,8,15,17,18].

The goal of the present study is to assess the health service needs and HIV risks of female (injecting and non-injecting) drug and alcohol users in two North-east Indian states (Manipur and Nagaland) in order to promote improved access to services. The specific study objectives were to: 1. Understand the local context of female drug and alcohol use in Manipur and Nagaland; 2. Describe the HIV risks among the female drug and alcohol users; 3. Identify the health and other problems experienced by these women; 4. Elucidate their health service needs; 5. Document the barriers to health service access; and 6. Recommend strategies for current health services in Manipur and Nagaland to improve access for female drug and alcohol users. This paper reports on the findings related to objectives 3-6.

### The context of Manipur and Nagaland

Manipur and Nagaland are two North-east Indian states that consistently report a high HIV prevalence, and in the case of Manipur, the highest in the country (adult HIV prevalence in 2009 was 1.4% in Manipur and 0.8% in Nagaland) [19]. The North-east region is characterised by various long-standing civil insurgent movements, deeply felt social conservatism, and substantial under-development. The Christian church is powerful in the public and private spheres in both states (especially in Nagaland), and as such, gives shape to socio-cultural values, as well as individual world views [20].

The sale and consumption of alcohol is illicit in both states, but relatively commonplace nevertheless. An estimated 1-2% of the adult population has injected drugs, mostly heroin and Spasmoproxyvon (SP, a pharmaceutical agent containing dextropropoxyphene that is designed to be taken orally, but also misused by injection) [21], with the result that IDUs have been the primary focus of HIV prevention interventions to date, and most IDUs are male. However, there is increasing recognition of the important contribution that sexual transmission is making to the spread of HIV in the region, especially in Nagaland, where the prevalence of HIV in 2008 was only 3.2% among IDUs but 14.6% among FSWs. The HIV prevalence among IDUs in the neighbouring state of Manipur is much higher at 28.6% (Personal communication from National AIDS Control Organisation, Northeast India). This major difference in HIV prevalence is explained by the very different patterns of drug use (type of drugs used and methods of use) in the two states.

Initially we planned a study of the health service needs of female injecting drug users, but were urged by local NGO staff to broaden the investigation to also cover non-injecting and alcohol users as their vulnerability was an area of concern for field staff that had not been well documented. The HIV prevention response in these states is being led by the National AIDS Control Organization (NACO) and the respective State AIDS Control Societies (SACS). Working alongside these government agencies since 2004 is Project ORCHID, which is funded by Avahan (Bill & Melinda Gates Foundation in India) to contribute to HIV prevention in selected districts of Manipur and Nagaland [22].

## Methods

### Study design

This qualitative study involved semi-structured, in-depth interviews with key informants (KIs) and focus group discussions (FGDs) with female and male drug and alcohol users in Manipur and Nagaland. The data collection took place in late 2009 and early 2010. The study was facilitated by partnerships with local Project ORCHID NGOs working in both urban and rural settings. These NGOs have existing relationships with female drug and alcohol users through their networks of outreach workers and peer educators, and the drug and alcohol using participants were generally recruited by these workers. Local research officers (ROs) were trained and supervised by the study investigators to coordinate and conduct the data collection in each state.

A female drug or alcohol user was defined as a woman who judged herself to be a regular (injecting or non-injecting) user of one or more of the following drugs in the past six months: alcohol, heroin/brown sugar, propoxyphene/Spasmo-proxyvon (SP), cannabis or amphetamine type substances (ATS). All participants were aged ≥ 18 years.

### Data collection

#### In-depth interviews with key informants (KIs)

A total of 27 KIs were purposively recruited and interviewed (15 in Manipur and 12 in Nagaland). The KIs were from a range of government, private and NGO services, and included: directors, program managers and field workers from organizations that work with sex workers, drug users, and vulnerable women; workers from drug detoxification and rehabilitation centres; HIV testing counselors; nurses working with HIV patients; a journalist; and a ‘booze joint’ owner (booze joints are illicit bars). Each interview took approximately one hour.

#### FGDs with drug and alcohol users

Five focus groups discussions (FGDs) were conducted with women alcohol and drug users in Manipur (2 in Imphal, 3 in Churachandpur), and four in Nagaland (3 in Dimapur, 1 in Wokha). There were 7-8 participants in each group, with a total of 39 participants in Manipur and 32 in Nagaland. Some groups included alcohol users exclusively, while others were a mixture of alcohol users, opiate users and those who used both substances. One FGD was conducted with male drug users in Imphal, Manipur (n = 11) and one in Dimapur, Nagaland (n = 12). Male drug users were considered to be another type of key informant regarding female drug and alcohol use. All FGDs were conducted by the local ROs, and each FGD lasted approximately two hours. FGD participants were identified through snowball sampling. The outreach workers approached potential participants in the field through their own networks, and in venues such as ‘booze joints’ and hotspots for drug use. The initially recruited participants were invited to bring along some of their peers. This resulted in a mixture of participants i.e. some were NGO service users and others were not, and while some were engaged in sex work, the majority were not. FGD participants were provided with payment to compensate for child care and travel costs.

Both the KI interviews and FGDs were conducted in the local language by the ROs using semi-structured interview guides. The thematic areas covered by the guides included: the local context of female drug and alcohol use; drug and alcohol use patterns and risk behaviours; current patterns of service usage; barriers and facilitators to service usage; perceived health needs; and expressed service needs. The interview guides were developed with the literature and the study objectives in mind, then refined and piloted in collaboration with the Indian partner NGOs and local research team members. They were translated into the local languages (Paite, Manipuri and Nagamese) after detailed discussion of the intended meanings and appropriate language for each thematic area. All interviews and FGDs were digitally recorded, transcribed, and translated into English for subsequent analysis.

### Data analysis

The data were analysed thematically [23]. A step-wise description of this process is provided below:

1. The interview and FGD transcripts were transcribed and translated by the field-based research officers under the supervision of two of the authors (CHS, CZS).

2. The printed transcripts were read over completely by the first author (MK) without any active coding at that stage.

3. All data were initially manually coded by the first author (MK) according to themes deductively derived from the interview guides. Initial themes were: patterns of drug and alcohol use; reasons for drug and alcohol use; HIV risk behaviours; health problems experienced by the women; other problems experienced by the women; differences between male and female drug and alcohol users; barriers to service access; and health and other service needs.

Subthemes were subsequently inductively identified for each theme through recognition of consistent patterns inherent in the data. Some sub-themes were further open-coded where appropriate. The sub-themes relevant to this paper are summarized below:

Health problems

drug & alcohol withdrawal; mental health; sexual & reproductive health; gastrointestinal problems; drug overdose; other.

Other problems

social exclusion; care of children; financial problems; lack of basic needs; violence.

Barriers to access

stigma & discrimination; financial constraints; poor/absent health infrastructure; limited geographical access; fear of identification as a high-risk group member.

**Health and other serviceneeds**

women only health services; detoxification & rehabilitation; mental health care; sensitization of health care workers; access to medicines; assistance to meet basic needs; other.

4. The findings were then synthesized, interpreted and reported following the structure provided by the study objectives.

5. Finally, the findings were presented to stakeholders in the two states in the context of dissemination meetings that included those who had participated in data collection, and feedback on the findings was actively sought.

When undertaking the data analysis and reporting the findings, the subjective perspectives of the female drug and alcohol users were privileged over those of the KIs. However, the perspectives of the KIs tended to support and augment what the FGD participants had to say, and are also presented here. We indicate when the views of the KIs are different from the women’s.

### Ethical issues

All potential participants were informed about the nature and purpose of the study when they were invited to participate. Those who volunteered to participate gave informed consent, and were assured of confidentiality. The staff who recruited the participants used a written plain language statement to guide the information they shared verbally with the women when inviting them to participate in the study. The women who agreed to participate were subsequently consented verbally using a format approved by the two ethics committee’s that reviewed the application – this involved the research officer signing that she had shared the required information with each of the women, and that they had agreed to participate – this process was witnessed whenever possible. Ethics approval was obtained from the University of Melbourne Human Research Ethics Committee and the Institutional Review Board of the Emmanuel Hospital Association, New Delhi, India.

## Results

### Background information on participants

The demographic information of all FGD participants are summarized in Table 1. The proportion of widowed and divorced women was very high considering the relatively young age of the participants.

**Table 1:** **Demographic information for allFGD participants**

**Variable**	**Manipur**		**Nagaland**	
	**Female**	**Male**	**Female**	**Male**
	**n = 39**	**n = 11**	**n = 32**	**n = 12**
**Mean age (range)**	31 yrs (20-45)	33 yrs (20-42)	28 yrs (18-38)	29 yrs (24-35)
**Schooling**				
- None	18%	0%	22%	0%
- Not completed	61%	18%	62%	50%
- Completed	21%	82%	16%	50%
**Marital status**				
- Single	15%	45%	34%	83%
- Married	21%	55%	31%	17%
- Widowed	31%	0%	13%	0%
- Divorced	33%	0%	22%	0%
**Drug/alcohol use**				
- Alcohol	90%	90%	91%	33%
- Heroin	64%	100%	0%	0%
- SP	26%	63%	16%	75%
**Children**	64%	45%	63%	17%

### Physical health problems

The most pressing health problems identified by these women were explicitly and implicitly associated with their dependence on drug and alcohol use. Many of the women spontaneously mentioned the symptoms of withdrawal from drugs and alcohol as their major health concern, and consequently associated the use of drugs or alcohol with feeling better.

"P1: People who use drugs are okay. They experience sickness only when they don’t have their daily dose. Without it we can’t even move, when people talk nicely to us, we take it very negatively. We face no health problems when we are on drugs. When we are high on drugs, we become smarter."

"P2: We are sick only when we don’t do drugs. Without our daily dose, we experience body aches… and start fighting with our husbands and children… but when we are on drugs, we are relieved of all the body aches and when we are not on drugs, the sickness increases. (FGD1 Dimapur, Nagaland)"

"P: Rice beer users also experience shaking of hands if they don’t drink. We feel scared when not drinking. (FGD2 Dimapur)"

For these women their health problems were associated with the absence of drugs or alcohol, rather than the presence of them. This is a different construct from that of the KIs who mostly perceived the presence of drugs and alcohol as the source of poor health for users.

A number of reproductive health problems were reported including unwanted pregnancy leading to both abortion and birth of children, white vaginal discharge, irregular or absent menstruation, and STIs – syphilis and gonorrhoea were named in particular. When the women suspected they had an STI, they often tried to self-treat rather than suffer the embarrassment of seeking diagnosis and treatment from a health professional. Long periods of amenorrhea were identified by the women using heroin in particular, and irregular menstruation contributed to late detection of pregnancy. In some cases of pregnancy, the identity of the father was difficult to ascertain, or the father refused to acknowledge paternity, with the result that some women were single parents to their children.

A lot of the participants, both FGD participants and KIs, identified a range of gastro-intestinal complaints as problematic for female alcohol users in particular, especially gastritis, stomach problems, liver problems, hepatitis, lack of appetite, weight loss due to poor food intake, diarrhoea, constipation, and nausea and vomiting. Drug overdose was mentioned as a health risk by many participants and some of the FGD participants provided graphic descriptions of their friend’s and their own experiences of overdose. Much less commonly mentioned were HIV, TB, and HCV.

### Mental health problems

Depression, stress and tension were frequently mentioned as a problem for women who use drugs and alcohol. Loss of hope was clearly expressed by some participants – many were resigned to their situation and could see no means of escape, and therefore, no viable future for themselves.

"P: No women who drink and do drugs are happy with their lives. (FGD2 Dimapur, Nagaland)"

"KI: If we look at their mental status, their motivation towards life and hope is very low. They are living for the sake of living. Once they die, then it is over, this is what they think. They don’t have any hope for the future. Being women, they also want to be a part of family where there are children, a husband and all. But it is only a dream to them. Sometimes we ask them “What is your wish for your life?” They answer “To live a life in a family like before” - this is their wish in life… They don’t have any hope for living a normal life. I think it might be the reason why they don’t want to go for health treatment or anything else. They think that even if they were free from drugs, they don’t have any place to go for a living. (KI3 NGO Project Manager, Manipur)"

### Social and economic problems

The women drug and alcohol users in this study experienced a number of social and economic problems including social exclusion, violence, concerns about children, and financial difficulties resulting in lack of basic needs such as food, health care and medicines. These problems were often of much greater concern to them than their physical health problems.

Social judgment and consequent exclusion, including rejection by families were commonplace features of these women’s lives, and clearly a major source of distress for them. Family abandonment preceded drug and alcohol use for some of the women, and was therefore perceived to be a cause of it, while for other women, family abandonment was a direct consequence of their drug and alcohol use. Sometimes the women deliberately stayed away from their families because they felt ashamed of their situation. Exclusion from the church was also frequently mentioned, and feelings of self-stigmatization and guilt were evident.

"P: Due to our No. 4 [heroin] use we have guilty feelings and are scared of our family. Even if we go home our family doesn’t want us to be at home, due to our drug habit. (FGD4 Churanchandpur, Manipur)"

"P: Due to alcohol use we do not want to participate in church or social gatherings. We prefer to be with our friends – we often drink together and we are scared to enter the church building. There is the feeling that they are staring only at us, so we are keeping aloof from the society and church. (FGD5 Churachandpur)"

Several of the women in this study provided vivid descriptions of being subject to emotional and physical violence, including rape. The source of violence included families, husbands, army, police, pressure groups (women’s and youth groups as well as underground groups) clients, and *chiltus* (local gangsters). The violence sometimes took the form of social humiliation e.g. the women were tonsured and publically scolded for the perceived immorality of their lifestyle.

"P: I have faced many hardships as I need money for No. 4 [heroin]. Male customers invite us and say they will pay the money at the place, and when we go to the place they have lied to us, and they rape us and will not give even 25 paise, and then they will run away. (FGD4 Churanchandpur, Manipur)"

"KI: They are facing a lot of violence. They get caught by some strange men in the hot spots and are having sex forcibly - they don’t get any money but get beaten up by these men. Very recently one incident happened where they were caught by some men who inserted a stick inside their private parts. It is very hard to hear about such inhuman behaviour, but it is common among the women who are chronically dependent on drugs and doing sex work. Who will complain to police for their sake? No one complains. If it happened to an ordinary housewife people would come out in support. Community people don’t like them. (KI8 NGO Project Manager, Manipur)"

According to participants, the women who use drugs and alcohol could themselves be perpetrators of violence when under the influence of alcohol in particular, and sometimes children were the target of their violence. The situation for some children of women who are drug and alcohol dependant was described as precarious, especially if the father is also a substance user or is absent. Several participants related instances of child neglect and mistreatment when children remained with their mother. This neglect took the form of poor supervision, inadequate provision of food, and limited access to education.

"P: Sometimes, when I take my daily dose and am high on drugs, I can teach my children very well. But sometimes when I don’t take my dose and experience turkey [drug withdrawal] I get mad and put that frustration onto my child and instead of taking care, I hit the child. When my thoughts are not much on getting my drugs I can teach the child, but when my thoughts are all focused on where and how to get my dose, I forget about my child. (FGD3 Dimapur, Nagaland)"

Some children were being cared for away from their mothers by family members or in orphanages, and this was described as a major source of guilt and pain for their mothers. The women’s inevitable financial difficulties led to many other problems such as lack of shelter, lack of food, and inability to provide not only for themselves but also for their children.

### Barriers to accessing health care

A number of barriers to accessing health services were identified by both the KIs and the women drug and alcohol users. The women’s own sense of shame, which was often deeply felt, or their reluctance to self-identify as a drug or alcohol user coupled with actual and anticipated discrimination from health care providers meant that they did not generally attend health care services. When the women experienced a health problem that could not be ignored, they mostly consulted with a pharmacist or a health worker from an NGO clinic. They generally avoided the government services because they feared being treated poorly, and the private services because they could not afford to pay.

Even attending the NGO services was sometimes problematic, as to do so could result in the woman being identified as HIV positive, a drug user or a sex worker (whether or not she was). The women understood that the NGO services were only available for women who were HIV positive, injecting drug users, or sex workers, and thus precluded non-injecting drug users and alcohol users.

"P: Injecting users are availing the services but oral users who are hidden do not want to avail the services because they do not want others to know they are taking drugs."

"F: What would be the reason that prevents them from accessing the services and programs they need?"

"P: They do not admit to themselves that they are an addict – they are hidden. Some do not want others to know about their drug use. Saying it is a way of self-disclosing our identity… In spite of telling our friends about the available services they do not listen to us. People have an idea that the services are only for positive people. (FGD3 Churachandpur, Manipur)"

"Another perceived short-coming of the NGO services was that the range of services offered was limited mainly to the provision of condoms, needles and syringes, whereas the women’s most pressing problems were unrelated to HIV prevention, and often beyond the scope of services offered by the HIV prevention NGOs. These problems included family conflict, social exclusion, mental health problems, drug and alcohol dependence, and financial difficulties."

"P1:They conduct group discussions with us. As part of the program, syringes and condoms are also available. No other programs except group discussions [they all laugh]."

"F: Can anyone tell about other services?"

"P2: Nothing, except syringes and condom distribution."

"F: Excluding syringes and condoms, any other program you access?"

"P1: No. With four to five participants they often conduct group discussions where they talk on the topic of prevention [laughs]. We are fed up of hearing and listening to the same topic which we have known, we already know [all of them laugh]."

"P2: We hesitate to attend the meeting assuming that the same message will be given."

"P3: We know about STIs and HIV. (FGD3 Churachandpur, Manipur)"

Other barriers included the cost of services and medicines, the inability of the women to travel away from their own locality, and poorly functioning or absent health services.

"KI: Some of the women say ‘We go to the hospitals but the doctor was not there, nurse was not there, and there was no medicine’. So if at the first shot, first time they go, if they don’t meet the doctor they don’t want to go the second time, they feel very discouraged, so lots of motivation is required. We tell them to go and they say ‘We’ll go, we’ll go’, but they don’t go. Yeah, access is one thing, plus facility availability in the hospital is also another thing. If everything is always available I’m sure they will be motivated to go, but many times they don’t get what they need and they are asked to buy [medicines], and they don’t have money to buy. (KI2 NGO Director, Nagaland)"

### Health and other service needs

#### Women-only health services

An integrated health service for women, staffed by women, was seen by the majority of participants as an important strategy for increasing access to care for female drug and alcohol users.

"KI: And there should be more women specific health centres – I don’t know whether it would be possible or not, but if there could be more health centres specifically for women irrespective of whether they are drug or alcohol users, for women in general. Any women can just walk into the centre and can get services. If something like that can be tried out I am sure it will go a long way in improving the health status of our women in general and maybe even the drug users in particular. (KI2 NGO Director, Nagaland)"

Related to the concept of women-only health services was that of women-only drop-in-centres (DICs) at the NGOs, as the current DICs are very male-dominated, and this actively deters the participation of women.

"KI: The women often say they feel uneasy to pass through the DIC and go for treatment as the males often tease them, and even if they did not tease them, they still feel uncomfortable because it is male dominated. (KI13 Outreach Worker, Manipur)"

The participants envisioned a women-only DIC as a place to rest, have tea and snacks, with make-up and other toiletries as well as bathing and clothes washing facilities. Another suggestion was the appointment of more female outreach workers and peer educators as part of the targeted HIV prevention programs. Legal advice services, children’s services, nutrition programs, and community mobilization programs were also mentioned as desirable services by the KIs in particular.

#### Detoxification and rehabilitation services for women

The women frequently emphasized the desperate need for women-only and women-friendly drug and alcohol detoxification and rehabilitation centres that are low cost and can accommodate children. They were also adamant that some sort of short-stay home/half-way house accommodation was necessary following completion of the rehabilitation program to reduce the very real risk of relapse, especially as women are not so easily reconciled with their family compared to men, even when recovered. The transition from rehabilitation to social reintegration was seen as a very vulnerable time for these women. Another important component of rehabilitation mentioned by many participants was meaningful vocational training, and access to micro-finance opportunities to start up small businesses.

"P: A short stay home [is needed] if the course in the rehab centre is short. After the detox period, vocational training would be very helpful for us. After completing the rehab course there should be something that can fully occupy our minds, this way it will be easier to forget what we desire. If not, after the course of rehab, if we stay the same way, it is very easy to use it again. A rehab centre is what we need in Churachandpur. (FGD3 Churachandpur, Manipur)"

It may not be necessary to admit all women seeking detoxification to a residential program, as community based programs were an alternative model of care mentioned by some KIs.

#### Mental health care

The need for mental health care and counselling for women drug and alcohol users was particularly emphasized by many participants, both the women themselves and the KIs.

"P: A good doctor, a psychiatric doctor – most of us are using alcohol and drugs due to depression and negative thinking – to provide quality counselling. If counselling and medical treatment are provided I believe this may be more helpful than syringes and condom distribution. Due to depression we are half-hearted. A doctor to be available in the NGOs to help us psychologically. (FGD5 Churachandpur, Manipur)"

#### Sensitization of health care workers and the general community

The need to raise awareness of the health and other problems faced by women drug and alcohol users among health care workers and the general community was frequently mentioned. The women anticipated and sometimes received poor treatment when seeking health care, and this was a major barrier to access.

"KI: Particularly in the health set up, whether in the government sector or the private sector, or the NGO sector, I think the sensitivity towards women, the issues of women – gender sensitivity – I think that is one area we are very much lagging behind. A woman should be made comfortable even if she is into drugs or alcohol. I think there has to be a lot of education and sensitization on gender sensitivity – that is one area that can be improved upon. (KI2 NGO Director, Nagaland)"

#### Free access to medicines and general health check-ups

Several FGD participants were of the view that general health services, integrated with STI testing and treatment services, should be made available to the women for free. Some were quite strident about their need for free medicines to treat the symptoms they were experiencing, especially treatments for gastritis and liver problems, a range of vitamins and minerals, and ‘glucose drips’. Frequently the drugs and vitamins were identified individually by name, and at times even by trade names e.g. ‘Liv 52’. They complained that they were repeatedly given the same medicines when attending the NGO clinics and wanted a range of medicines to be available for them free of cost. Some identified the restricted access to medicines as a factor that deterred women from returning to the clinics.

"P1: What I want is, as most of us have a gastritis problem, we need medicine for liver, stomach. And there are some who are dying when they skip food for two to three days – for such persons, they need vitamin and glucose."

"P2: Mostly drug and alcohol users have gastritis problems. I am not criticizing the existing services, but when we access free medical check-ups, free medicine is not available for us. So a place where free medicine for liver, stomach, and glucose should be available. When I start drinking alcohol I can stay without food even for three months, and if I stop drinking I am dying, and after four to five glucose drips, I will be alright again. (FGD5 Churachandpur, Manipur)"

#### Assistance to meet basic needs

The need to help women drug and alcohol users find employment or livelihood opportunities was also mentioned. As many of the women had been rejected by their families, they often lacked a safe and secure place to sleep.

"P: We need a place to sleep as we drug user are busy hunting for money for our drug use, and most of the time we are not allowed to enter our own house. We need a proper place with food as we cannot sleep in the street, and this will also help us mentally with our trouble in finding a place to sleep. (FGD3 Churachandpur, Manipur)"

#### A place for engaging in sex work

Some of the FGD participants who were sex workers identified the need for a safe place where they can both live and sell sex.

"P: We need a house where we can also sleep and for sex work. Where one of the staff will play the role of manager, and with a clinic attached."

"P: A place to sell sex and earn money for IDU sex workers."

"P: The pimps are taking advantages of us, they disregard us and often cheat us, and they never pay the exact amount of our share."

"P: What you said is true. We have to share the money with the pimp fifty-fifty. So if we could have a place where we can sell sex and earn money. We can share fifty to the pimp manager. Fifty from the manager’s share should include house rent and food."

"P: If three or four or up to seven of us can stay in a place in such a way we could earn and live properly."

"P: This way we can stand as a strong self help group."

"(FGD 3 Churachandpur, Manipur)"

## Discussion

### Health, social and economic problems

The women drug and alcohol users in this study experienced a range of health problems including drug and alcohol dependence/withdrawal, a number of reproductive health complaints, and poor mental health. They also described many social and economic problems that clearly have the potential to impact negatively on their health and well-being, and on their capacity to access services, including financial difficulties, social exclusion, violence, and concerns about the well-being of their children. From the women’s perspective, their health problems were mostly over-shadowed by their social and economic problems.

The main health problems experienced by these women related to symptoms associated with withdrawal from drugs or alcohol, and as these symptoms were alleviated by the use of drugs or alcohol, the use of these substances made them feel ‘well’. This construct contrasts with that of most health care providers who are likely to perceive drugs and alcohol as a source of illness rather than wellness. However, even though the women frequently articulated this bodily experience of drugs and alcohol making them feel ‘better’, there was also a lot of discussion regarding the need for detoxification and rehabilitation services, clearly indicating that drugs and alcohol are also perceived, at least at some level, to be problematic in their lives.

Poor mental health is both a cause and a product of drug and alcohol dependence. Social exclusion and violence, instances of which were vividly described by the women in the FGDs, are also likely to have a negative impact on their mental health, as does rejection by and separation from families. A large study of women and drug use in India identified 75% of female substance users as having a possible underlying psychiatric disorder such as depression or anxiety, and one-third reported at least one suicide attempt during the previous year [8].

Considering the relatively young age of these women, a large proportion were widowed or divorced (almost two-thirds in Manipur and one-third in Nagaland). As in other parts of India, divorcees and widows are socially and economically vulnerable, and if young, sexually vulnerable as well. Young widowhood in Manipur in particular is likely to be associated with the death of a drug-using spouse from drug overdose or HIV. It is possible that for some of these women, divorce has been a consequence of their drug or alcohol use. However, it is also likely that the precarious situation of widows and divorcees is contributing to both drug and alcohol use and engagement in sex work.

Violence against these women was commonplace and not limited only to those who were engaging in sex work. Similarly, in the large Indian study mentioned above, 73% of female substance users had experienced violence, and of these, 91% reported physical violence, 53% sexual violence, and more than 75% had sustained physical injury as a result of the violence [8]. In South India, violence against sex workers was associated with economic insecurity, more adverse reproductive health outcomes, and greater risk of HIV and STIs [24,25]. It is clear that interventions to reduce the level of violence experienced by these women are greatly needed. However, while successful community empowerment of sex workers in South India has been able to reduce the amount of violence perpetrated against them by the police in particular [26], effective collectivization of FSWs in Manipur and Nagaland has not been very successful to date [12], and interventions targeting only sex workers will not help all of these women.

The situation for the children of women who use drugs and alcohol is concerning. These children can develop emotional, behavioural and learning problems, witness and be subject to violence, and become involved in substance use at a young age [8]. In Manipur and Nagaland, having children outside of wedlock is socially unacceptable and there are few support structures in place to assist women and children in this situation. Both the mother and the children are likely to suffer poverty and social exclusion as a consequence of single parenthood. Arguably, one of the best ways to support the children of women who are drug and alcohol users is to support their mothers, and one of the best ways to support their mothers is to help with their children. Programs that are trying to attract women drug and alcohol users should also consider the needs of their children, as has happened at the Sonagachi Project in the neighbouring state of West Bengal [27], and with the Ambar project in Venezuela [28].

### Health and other service needs

According to the participants in this study, the establishment of a comprehensive, integrated women’s health centre is more likely to attract women drug and alcohol users than the current HIV prevention services or existing government health services. Not only would this model provide a ‘one-stop shop’, it would also offer some protection from identification as a member of a high risk group. These centres are more likely to meet the needs of women if they are low-cost, strategically but discretely located, open at times that suited the clients, and staffed by a range of female staff, including female doctors. The range of health services could potentially encompass general health care, family planning, antenatal care, mental health, counseling, HIV testing, STI management, opioid substitution therapy, TB treatment, ART, overdose management, and condom and needle & syringe distribution.

One of the most pressing service needs from the perspective of the women was detoxification and rehabilitation. Services for women requiring detoxification and rehabilitation for alcohol addiction are currently not available, and the options for women drug users who want assistance to reduce their drug use are very limited. To the best of our knowledge, there are no Alcoholics Anonymous or Narcotics Anonymous type programs for women in either state. In the absence of women-friendly detoxification and rehabilitation services it is difficult to imagine how women who are dependent on drugs and alcohol can gain a measure of control over their lives.

Interventions that aim to promote the mental health and well-being of these women are potentially available, affordable, and effective [29], and may reduce risk-taking behaviours, and increase participation in HIV testing and adherence to treatments. Such interventions also have the potential to assist these women with anger management, and thereby reduce their own acts of violence [29].

Health care workers located at services that are likely to be patronized by female drug and alcohol users need to be sensitized to the health services needs of these women, and equipped to provide non-discriminatory treatment and care. This is especially the case for specialist obstetrician and gynaecologists, primary health care centres, mental health services, and hospital staff.

Awareness raising and sensitization of church leaders and groups are also important in the context of Manipur and Nagaland. The Church has a powerful influence over societal attitudes to these vulnerable women [20], as well as huge potential to promote social inclusion and provide them with much needed supports. A similar program among women’s and youth groups and police could also be beneficial.

The suggestion that FSWs be provided with a safe place where they can both live and work is a tantalizing one. However, there are obvious challenges to the establishment of such a venue in a very conservative setting where organised anti-sex worker campaigns are a reality and the legal status of sex workers is precarious. Nevertheless, it must be acknowledged that this type of arrangement has the potential to reduce the risks of HIV and STI infection and exposure to violence, as well as making it easier to target FSWs with health and other services. Many of these women are homeless, so it would also provide them with shelter.

### Limited overlap between the felt needs of the women and the services provided by HIV prevention programs

As women who use drugs and alcohol are reluctant to attend mainstream health services because they anticipate and often encounter discriminatory attitudes on the part of the health care workers, and they are unable to afford the service and the associated costs of tests and medicines, another point of entry into the health care system for these women is through the HIV prevention and care services provided by NGOs (funded mostly by government and large donor agencies), which were viewed as more sympathetic to their situation. Health care for HIV positive women, and HIV prevention services for women who inject drugs, FSWs and female sexual partners of IDUs are predominantly provided by NGOs in Manipur and Nagaland. The content of these services is determined by policy makers and program planners with an HIV prevention and care agenda firmly in mind e.g. ART treatment, HIV testing, STI treatment, IEC, needle/syringe and condom distribution. However, these services as currently structured are unlikely to meet the needs of the women participating in this study. This lack of congruence between the services the women say they want and the services actually provided probably, at least in part, accounts for the relatively poor uptake of these services by vulnerable women.

Women drug and alcohol users’ most pressing needs were often not directly related to health, and certainly HIV and STIs were not high on the list of issues that concerned them most. Financial insecurity, inability to adequately feed and shelter themselves and their children, family and social rejection, violence, and related to all of these, poor mental health, were the main concerns of the study participants. These findings resonate with those from the larger Indian study on women and substance use that ranked the top ten services that female substance users had not accessed but would like to as: mental health services, vocational training, housing/shelter, legal/advocacy services, nutritional programs for children, microfinance, female condoms, OST, de-addiction services for women, and STI services [8]. Additionally, these women perceived NGOs as the place to which they were most likely to turn for support [8]. Consequently, if NGOs who currently implement HIV prevention programs are able to expand their services so that they more effectively respond to the stated needs of these women, more women may attend. However, a major challenge for any NGO wanting to offer a more integrated service is the struggle to mobilise and coordinate sufficient funds from multiple agencies and government sectors in order to do so.

### Study limitations

This study has a number of limitations that should be considered when interpreting the findings. There could be some selection bias because the FGD participants were recruited through the NGO networks, but this is somewhat offset by the fact that we deliberately recruited both NGO service-users and non-users. Given the sensitive nature of the questions about socially taboo behaviours of women, some participants may have been inclined to provide more socially acceptable responses, at the expense of valid responses, resulting in bias. We were unable to identify those participants engaged in sex work from the majority who were not, and it is probable that the health service and other needs of women engaging in sex work are somewhat different from those who do not. It is a qualitative investigation so findings cannot be generalized, although there is no compelling reason to assume that the situation is very different for other groups of female drug and alcohol users in Manipur and Nagaland. A follow-up survey with a representatively sampled group of women would strengthen the findings.

## Conclusion

This qualitative study aimed to describe the health needs of women who use drugs and alcohol in Manipur and Nagaland in order to identify strategies for strengthening services so that they can more effectively reach and meet the needs of these extremely vulnerable women. The study findings highlight a range of factors that have direct implications for the provision of health and other services, including HIV prevention programs, and raise a number of key questions that need to be answered if the situation of women drug and alcohol users in Manipur and Nagaland is to be improved: How can HIV prevention programs attract vulnerable women, including women who inject drugs and engage in sex work, when the range of services they are funded to offer do not meet the expressed needs of the women? How can women who are dependent on drugs and alcohol gain access to effective and affordable detoxification and rehabilitation services in the absence of such services? Below are some suggested strategies and activities that NGOs providing HIV prevention programs can initiate in order to promote greater participation of women in their programs. A few NGOs in Manipur and Nagaland have already implemented some of these activities, often with very limited funding.

• Active recruitment of female staff members, especially among the cadre of peer educators.

• Provision of the following:

• Safe spaces for women that are welcoming and that encourage informal interaction between the women and the staff

• Basic needs such as food and shelter

• Programs for the children of vulnerable women

• Women-friendly OST services

• Basic health care and family planning services alongside STI services

• Mental health literacy training for all NGO clinic and field staff.

• Liaison with and sensitization of the following:

• Existing detoxification and rehabilitation services to promote the development of women-friendly services

• Health care workers in mainstream services including maternal and child health services

• Church and women’s groups interested in supporting vulnerable women

• Law enforcement services.

• Sustained advocacy with relevant government ministries, departments and agencies regarding the health and other needs of women drug and alcohol users.

In the long-term, the optimal model for addressing the questions raised above is probably the provision of integrated women-only services, but achieving this requires collaboration across sectors of government involving those responsible for health, HIV prevention, children’s welfare, drug and alcohol treatment, justice, employment and education.

## Competing interests

The authors declare that they have no competing interests.

## Authors’ contributions

All authors contributed to study conception and all have read and approved the final manuscript. MK and AD were responsible for study design, analysis and interpretation, and drafting the manuscript. CHS, CZS and TNJ contributed to acquisition of the data and interpretation of the findings.

## Pre-publication history

The pre-publication history for this paper can be accessed here:

http://www.biomedcentral.com/1471-2458/12/825/prepub
